# Chronic nicotine differentially affects murine transcriptome profiling in isolated cortical interneurons and pyramidal neurons

**DOI:** 10.1186/s12864-017-3593-x

**Published:** 2017-02-20

**Authors:** Jie Yang, Ai-Yi Liu, Bo Tang, Dong Luo, Yu-Jie Lai, Bing-Lin Zhu, Xue-Feng Wang, Zhen Yan, Guo-Jun Chen

**Affiliations:** 1 Department of Neurology, The First Affiliated Hospital of Chongqing Medical University, Chongqing Key Laboratory of Neurology, 1 Youyi Road, Chongqing, 400016 China; 2 Department of Physiology and Biophysics, State University of New York at Buffalo, Buffalo, NY 14214 USA

**Keywords:** Nicotine, Somatostatin, Interneuron, Pyramidal neuron, Transcriptome, Mitochondria

## Abstract

**Background:**

Nicotine is known to differentially regulate cortical interneuron and pyramidal neuron activities in the neocortex, while the underlying molecular mechanisms have not been well studied. In this study, RNA-sequencing was performed in acutely isolated cortical somatostatin (Sst)- positive interneurons and pyramidal neurons (Thy1) from mice treated with systemic nicotine for 14 days. We assessed the differentially expressed genes (DEGs) by nicotine in Sst- or Thy1- neurons, respectively, and then compared DEGs between Sst- and Thy1- neurons in the absence and presence of nicotine.

**Results:**

In Sst-neurons, the DEGs by nicotine were associated with glycerophospholipid and nicotinate and nicotinamide metabolism; while in Thy1-neurons those related to immune response and purine and pyrimidine metabolisms were affected. Under basal condition, the DEGs between Sst- and Thy1- neurons were frequently associated with signal transduction, phosphorylation and potassium channel regulation. However, some new DEGs between Sst- and Thy1- neurons were found after nicotine, the majority of which belong to mitochondrial respiratory chain complex.

**Conclusions:**

Nicotine differentially affected subset of genes in Sst- and Thy1- neurons, which might contribute to the distinct effect of nicotine on interneuron and pyramidal neuron activities. Meanwhile, the altered transcripts associated with mitochondrial activity were found between interneurons and pyramidal neurons after chronic nicotine.

**Electronic supplementary material:**

The online version of this article (doi:10.1186/s12864-017-3593-x) contains supplementary material, which is available to authorized users.

## Background

The inter-play between interneurons and pyramidal neurons in the neocortex forms the basis of inhibition and excitation and neural network function [1, 2]. Interneurons powerfully regulate pyramidal neuron functions, albeit with low synaptic inputs in number [3]. Recent studies demonstrate that GABAergic interneurons play a critical role in local circuit function and behavior [4–6]. Dysfunction of GABAergic signaling is associated with age-related cognitive decline, schizophrenia, ischemia and Alzheimer Disease [7–10].

Nicotine has been found to improve working memory and learning by activating nicotinic acetylcholine receptors (nAChRs) [11, 12]. Emerging evidence has shown that nicotine differentially regulates interneuron and pyramidal neuron activities. For instance, nicotine layer-specifically regulates neuronal activities where distinct interneurons and pyramidal neurons are located [13]. While nAChRs enhance AMPA receptor mediated current and firing rate in interneurons [14, 15], they cause a sustained reduction of NMDA receptor mediated currents in pyramidal neurons [16], suggesting an involvement of different molecular mechanisms. Although gene profiling studies have made great progress in identifying individual neurons in the cortex [17, 18], and microarray studies have demonstrated that nicotine causes different gene expression in neuroblastoma cell line [19, 20] and in distinct brain regions including the cortex [21, 22], how nicotine may specifically regulate the gene expression in interneurons in relation to pyramidal neurons in cortical circuit remains unknown.

In this study, we acutely isolated cortical somatostatin (Sst) labeled interneurons and thymus cell antigen 1 (Thy1) labeled pyramidal neurons in mice treated with saline and systemic nicotine for 14 days, and the transcriptome profiling was compared in Sst- and Thy1-neuons with or without nicotine. We found that in Sst- neurons the most prominent genes affected by nicotine were associated with glycerophospholipid and nicotinate and nicotinamide metabolism, and in Thy1- neurons those associated with immune response and purine and pyrimidine metabolisms were influenced. In addition, the differentially expressed genes (DEGs) between Sst- and Thy1-neuons were associated with mitochondrial respiratory chain complex after nicotine treatment.

## Methods

### Animals

The following mice were used throughout the experiments: GIN mice (FVB-Tg [GadGFP] 45704Swn/J, The Jackson Laboratory, #003718) expressing enhanced green fluorescent protein (eGFP) in a group of somatostatin-positive interneurons (Sst), and thy1-YFP-H (YFPH) mice (B6.Cg-Tg [Thy1-YFP] HJrs/J, The Jackson Laboratory, #003782) expressing yellow fluorescent protein in layer V pyramidal neurons (Thy1) [23, 24]. A previous study has compared the genetic profiling between four lines of mice including GIN and YFPH used in our study. The authors found that the correlation coefficient between the samples from these two mice was not significantly different, indicating that GIN and YFPH mice do not show significant transcriptome differences in specific cell types, under basal condition [17]. Although another major group of interneurons are parvalbumin (PV)-positive, they do not express nicotinic acetylcholine receptors in the cortex [25, 26] and thus were not chosen in this study.

Twelve male mice (20 g, 5–7weeks) were divided into four groups, with 3 GIN or YFPH mice in each group receiving nicotine or saline, respectively. Mice were implanted subcutaneously with an osmotic mini pump (model 2002; Alzet, Cupertino, CA) that delivered 48 mg/kg/d nicotine hydrogen tartrate (Sigma, St. Louis) or saline vehicle for 14 days [27, 28]. This dosage of nicotine is known to alter neuronal activity and gene expression in the brain of mice [29, 30]. Mice were housed in the 12/12 h light/dark cycle and were given food and water ad libitum. Genotypes were confirmed by PCR analysis before study. All protocols were approved by the Commission of Chongqing Medical University for ethics of experiments on animals and were in accordance with international standards.

### Brain slice preparation and cell sorting

Mice were deeply anesthetized with sodium pentobarbital (100 mg/kg; i.p.) and decapitated [30]. Brain slices (400 um) were prepared by a vibratome (Leica VP1200S, Germany) in ice-cold artificial cerebral spinal fluid (ACSF) containing (in mM):119 NaCl, 2.5 KCl, 1 MgCl2, 26 NaHCO3, 1 CaCl2, 1.25 NaH2PO4, 25 dextrose aerated with 95% O_2_/5% CO_2_ (pH7.4). The slices were then incubated in ACSF containing 50 μM APV, 20 μM DNQX and 100nM TTX for 30 min at 32 °C and then for at least 30 min at RT. The cortex were morphologically dissected and enzymatically digested by pronase E (1.5 mg/ml; Sigma Aldrich) in ACSF (containing 50 μM APV, 20 μM DNQX and 1 μM TTX) for 45 min, then triturated by three fire-polished Pasteur pipettes (400, 300 and 150 μm in inner diameter, respectively) in ACSF containing APV, DNQX, TTX and 1% BSA [31, 32]. The resultant pieces were allowed to settle for 2 min and the supernatant were collected for centrifugation at 800 g for 5 min, which removed the cellular debris and molecular contaminants [31]. Fluorescent cells (Sst- or Thy1- positive) were carefully aspirated by a micropipette (30–50 μm) shaped by a pipette puller (P-97, Sutter, USA) [32]. 10 cells were aspirated each time and 30 cells were collected from each mouse. Cells (10 in each) were then transferred to 3.5 μl lysis buffer containing 2% Triton X-100 and 5% RNaseOUT (20U/μl, Invitrogen) in nuclease free water and were immediately stored at − 80 °C. A total of 90 cells from 3 mice in each group were collected for final sequencing. This amount of cells has been proven to be enough to reliably measure the population transciptome [17].

### RNA library construction and sequencing

SMARTer Ultra Low Input RNA for Illumina Sequencing kit (Clontech) was used to amplify cDNA from cell lysates, according to manufacturer’s instruction. This kit allows high-quality cDNA synthesis in a single cell containing as low as 10 pg RNA [30, 33]. The cDNA library was generated using Ovation® Ultralow DR Multiplex System (NuGEN). The amplified products were quantified by Qubit® 2.0 Fluorometer (Invitrogen) and validated by Agilent 2100 bioanalyzer (Agilent). TruSeq PE Cluster Kit v3 (Illumina) were used for cluster generation in an Illumina cBOT instrument. And the sequencing was performed on an Illumina HiSeq2500 instrument (Illumina) following the manufacturer’s protocol. Raw reads were performed with base-calling and quality-filtering process and then aligned to mouse genome (version: mmu10.p2) using the Tophat program [34]. Differentially expressed genes (DEGs) were expressed as FPKM (Fragments Per Kilobase of exon model per Million mapped reads), which were filtered by EBSeq algorithm, after the significant analysis and false discovery rate (FDR) analysis under the following criteria: i) Fold Change > 2 or <0.5; ii) FDR < 0.05, in which the FDR was used to correct P values [35–37]. Gene ontology (GO) annotations from NCBI, UniProtand the Gene Ontology were used to elucidate the biological implications of DEGs [38]. Pathway analysis was used to find out the significant pathway of DEGs according to KEGG (Kyoto encyclopedia of genes and genomes) database [39, 40]. Fisher’s exact and FDR tests were used to select the significant GO categories and pathways, and the threshold of significance was defined by *P*-value and FDR [41, 42]. The pathway and gene interaction networks were built based on KEGG database [43, 44].

### Semi-quantitative PCR

Reaction mixtures contained of 5 × buffer (contained 2 mM MgSO4), 0.5 mM each of the dNTPs, 1 μM primers, 0.02U Phanta® Super Fidelity DNA Polymerase (Vazyme, Nanjing, China), and 0.5ul of the cDNA template was made from the SMARTer Kit. The thermal cycling program for the amplification was as follows: 95 °C for 3 min, 45 cycles of 95 °C for 30 s, 56 °C or 60 °C for 15 s, and 72 °C for 15 s followed by 72 °C for 7 min. The triplicate PCR products were mixed and visualized in a 1.5% agarose gels. The expression levels were visualized by a chemiluminescence system (Fusion Fx7, Fisher Biotech) and quantified by Quantity One software (Bio-Rad, CA, USA). Data were shown as means ± SEM (*n* = 3). The statistically significant differences between groups were accessed by paired Student’s *t* test using Graphpad Prism 5 software (GraphPad). Detailed sequences of the primers were shown in Additional file 1: Table S1.

## Results

### Overview of RNA sequencing

All samples were subjected to massively paralleled paired-end cDNA sequencing. Before the read (sequencing fragment) mapping, clean reads were obtained from the raw reads (5GB) by removing the adaptor sequences from each library, reads with >5% ambiguous bases (noted as N) and low-quality reads containing more than 20% of bases with qualities of <20. Of all uniquely mapped reads, about 60% were aligned to the transcript exon, 10% at the intron, 25% at the UTR regions and the remaining at TES (transcription end site), TSS (transcription start site) and intergenic regions (Additional file 2: Figure S1A). Mapped reads (Additional file 1: Tables S2 & 3) were distributed consistently on the chromosomes (Additional file 2: Figure S1B-E).

To identify the purity of manual sorting, we measured the expression level of genes associated with non-neurons, such as glia, astrocyte, oligodendrocyte, microgila and red blood cells [17, 18, 45]. Generally, non-neuron marker genes such as glia marker Vim, astrocyte marker Gfap and red blood cell marker Hbb-b1 in control groups were expressed at very low level (Additional file 1: Table S4).

### Nicotine induced DEGs related to different pathways in Sst- and Thy1-neurons

**Fig. 1:**
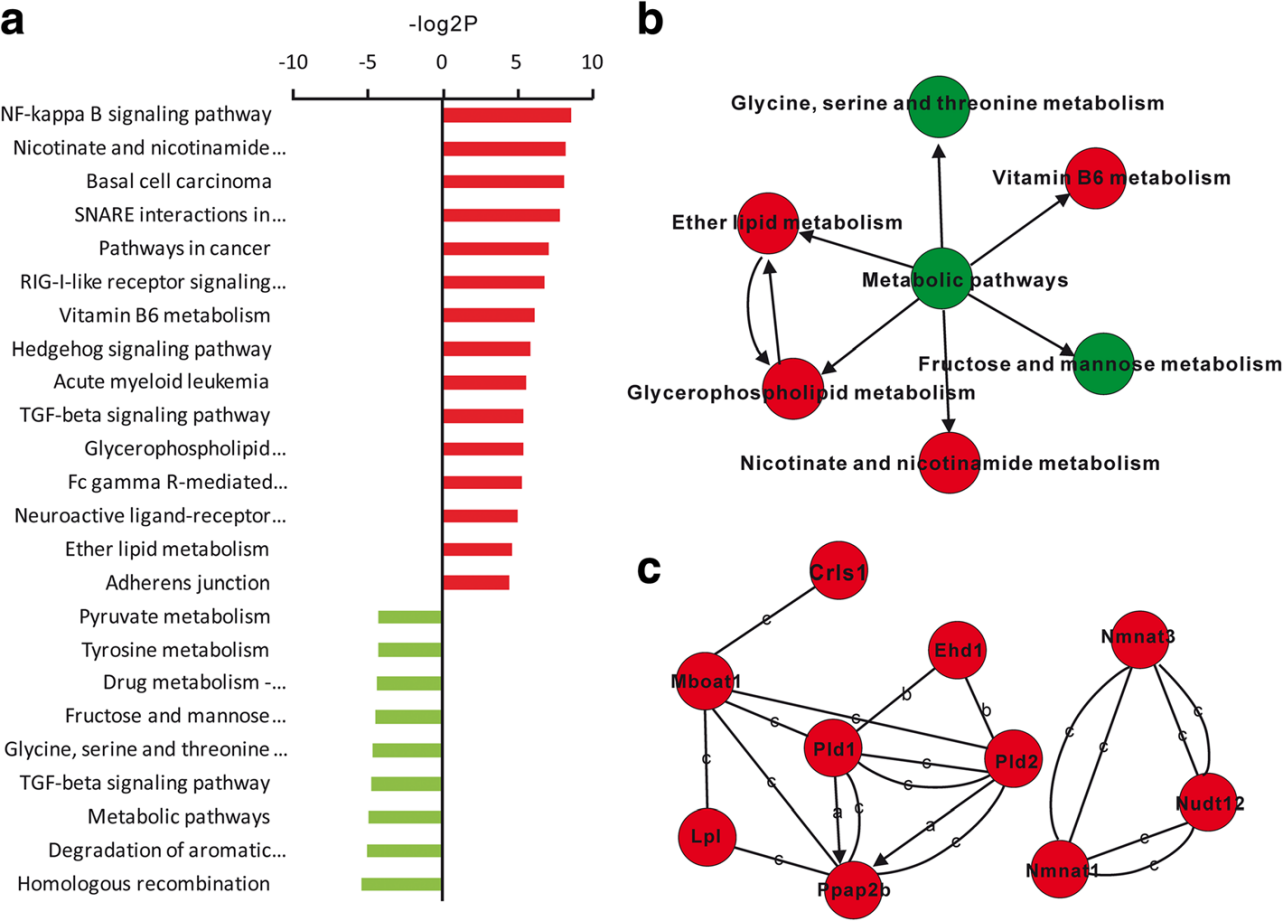
Pathways and functional networks influenced by nicotine in Sst-neurons. **a** Up- and down-regulated pathways affected by nicotine. **b** Interaction network of metabolism related pathways. *Red* and *green* circles represent significantly up- and down- regulated pathways by nicotine (*P* < 0.05), respectively. **c** Network of up-regulated genes involved in glycerophospholipid (*left*) and nicotinate and nicotinamide (*right*) metabolisms after nicotine treatment. Pld1, Pld2 and Paap2b are highly-connected genes with a central role in the network than peripheral genes. c, compound; b, binding/association; a, activation

There were 789 and 711 DEGs (>2 fold change; FDR < 0.05) in Sst- and Thy1-neurons after nicotine treatment, respectively. Additional 20 common genes shared by Sst- and Thy1-neurons were both up- or down- regulated by nicotine (Additional file 3: Supplemental Excel S1). Pathway interaction network analysis revealed that 24 pathways were affected by nicotine in Sst-neurons (Fig. 1a, Additional file 4: Supplemental Excel S2). Not surprisingly, nicotine significantly activated NFκB signaling as previous reported [46]. The genes associated with cancer and neuroactive ligand-receptor interaction were upregulated while those in metabolic pathways were downregulated by nicotine (Fig. 1a). As shown in Fig. 1b, an integrated pathway tree was depicted according to KEGG database, in which hyperactive pathways associated with glycerophospholipid metabolism and nicotinate and nicotinamide metabolism (red) and hypoactive metabolic pathways (blue) were shown. Ppap2b, Pld2, Pld1, Mboat1 and Lpl were grouped into glycerophospholipid metabolism, and Nmnat3, Nudt12 and Nmnat1 were categorized into nicotinate and nicotinamide metabolism, which were all activated by nicotine (Fig. 1c). Among these genes, Pld1, Pld2 and Paap2b owned the strongest degree of centrality, suggesting that glycerophospholipid metabolism has a powerful role in Sst- neurons after nicotine.

**Fig. 2:**
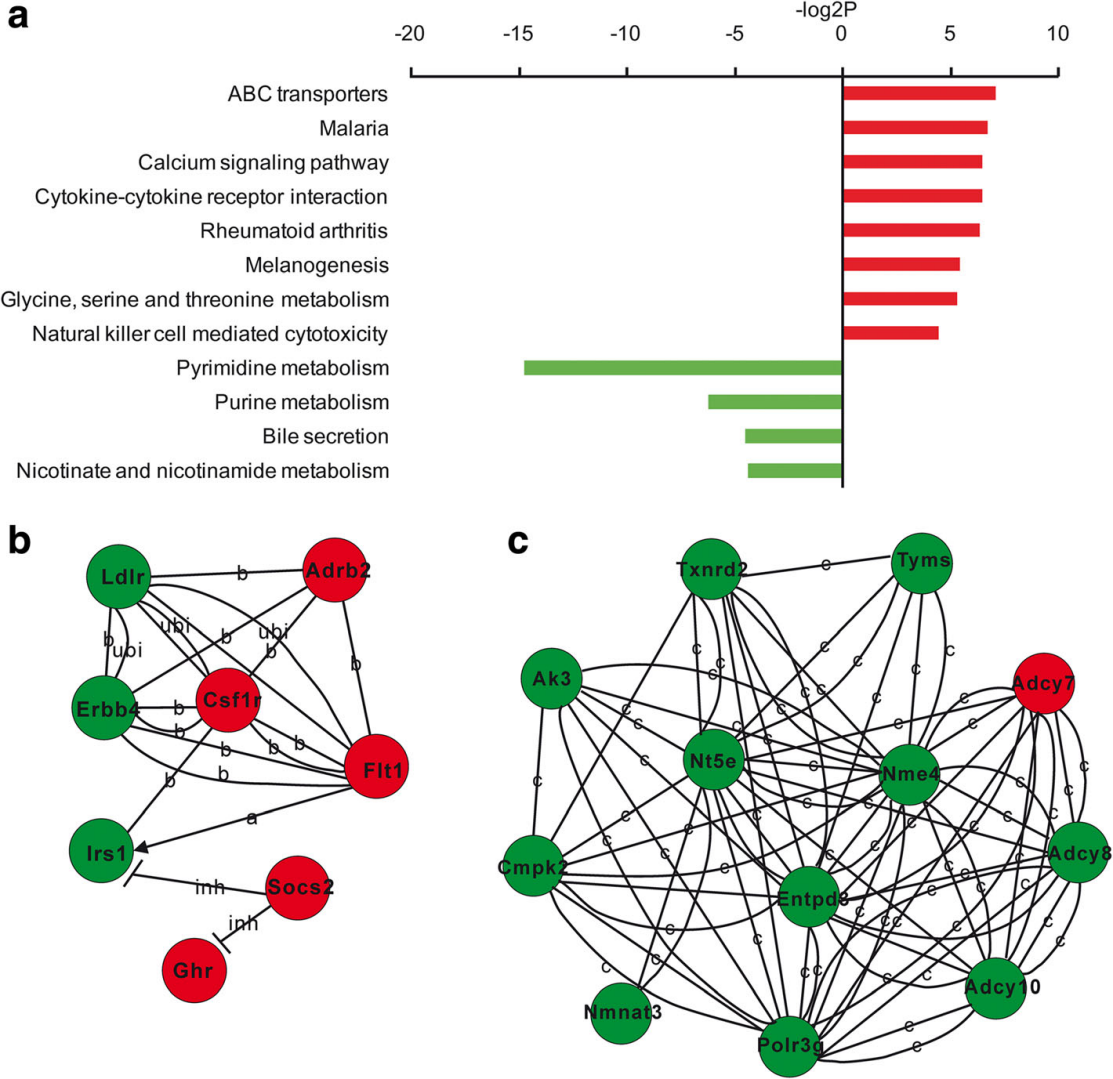
Pathways and functional networks affected by nicotine in Thy1-neurons. **a** Up- and down-regulated pathways affected by nicotine. **b** Network of genes involved in cytokine-cytokine receptor interaction and calcium signaling pathway. **c** Network of genes involved in the metabolism of purine and pyrimidine. *Red* and *green* circles represent significantly up- and down- regulated genes by nicotine (*P* < 0.05), respectively. Csf1r and Flt1 (**a**), and Nme4 and Nt5e (**b**) are highly-connected genes with a central role in the network. c, compound; b, binding/association; inh, inhibition; ubi, ubiquitination

In Thy1-neurons, nicotine affected 12 pathways related to ABC transporters, calcium signaling, cytokine-cytokine receptor interaction, rheumatoid arthritis, natural killer cell mediated cytotoxicity and pyrimidine and purine metabolism (Fig. 2a, Additional file 5: Supplemental Excel S3). It was interesting to note that pathways in cytokine-cytokine receptor interaction, rheumatoid arthritis, and natural killer cell mediated cytotoxicity are all associated with immune response. According to KEGG database, gene networks related to immune response (Fig. 2b) and pyrimidine and purine metabolism (Fig. 2c) were built. Among these genes, Csf1r and Flt1, Nt5e and Nme4 were considered as the highly-connected genes in immune response (Csf1r and Flt1) and pyrimidine and purine metabolism (Nt5e and Nme4), respectively. These results indicated that in Thy1-neuons, nicotine significantly increased immune response genes while decreased pyrimidine and purine metabolism related genes.

### DEGs between Sst- and Thy1-neurons under basal condition

**Fig. 3:**
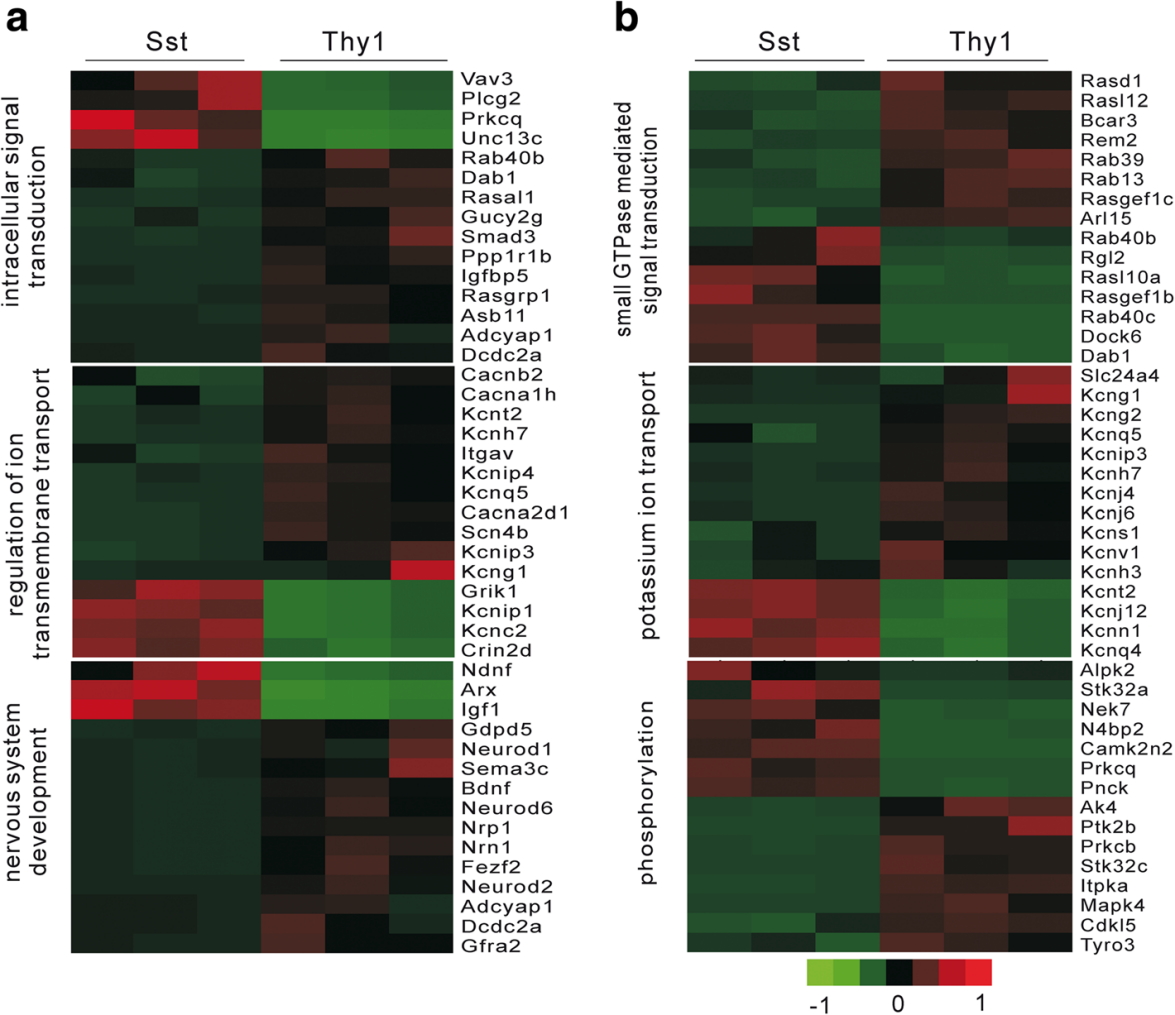
Top-ranked GO terms associated with DEGs between Sst- and Thy1-neurons under basal condition. **a** Heat map representation of the top 15 genes with the lowest FDR involved in intracellular signal transduction, regulation of ion transmembrane transport and nervous system development, respectively (*n* = 3). **b** Representative genes involved in small GTPase mediated signal transduction, potassium ion transport and phosphorylation, respectively (*n* = 3). Gene expression is shown with pseudocolor scale (−1.0 to 1.0) with *red* denoting high gene expression levels and *green* denoting low gene expression levels. FDR is ranged from a maximum of 1.11 × 10^−15^ to 0, which is used for the correction of *P* values

There were total 3185 DEGs between Sst- and Thy1-neurons without nicotine treatment (Additional file 6: Supplemental Excel S4). GO analysis revealed that these DEGs were significantly enriched in intracellular signal transduction (*P* = 4.4 × 10^−9^), nervous system development (2.16 × ^−7^) and potassium ion transport (2.66 × ^−6^). Small GTPase mediated signal transduction (*P* = 7.8 × 10^−7^) and phosphorylation (*P* = 3.7 × 10^−6^) were also significant (Additional file 7: Supplemental Excel S5). The representative genes with the lowest FDR involved in these biological processes were listed in Fig. 3. As previously known that nervous system development genes such as neuronal differentiation 1/2/6 (Neurod1/2/6) were associated with glutamatergic neuron development in axon outgrowth and glutamatergic synaptogenesis [47, 48]. Moreover, potassium ion channel subtype genes Kcng1, Kcng2, Kcnq5, Kcnip3, Kcnh7, Kcnj4, Kcnj6, Kcns1, Kcnv1 and Kcnh3 were significantly higher in Thy1-neurons, while Kcnt2, Kcnj12, Kcnn1 and Kcnq4 were significantly higher Sst-neurons. Some of the DEGs might be cell-type specific. The genes with high expression pattern (FPKM >1000) relative to low expression pattern (Fold change >20, FDR = 0) were considered as specific genes [18]. The top 80 Sst- and Thy1-neuron specific genes were shown in Additional file 8: Figure S2. Many of these were expressed in a manner consistent with previously published observations [17, 45], while some genes such as Thsd7a in Sst-neurons and Kcnh7 and Crym in Thy1-neurons were not previously reported, suggesting that these genes might represent new marker genes in these neurons.

### Pathway analysis of DEGs between Sst- and Thy1- neurons in the absence and presence of nicotine

**Fig. 4:**
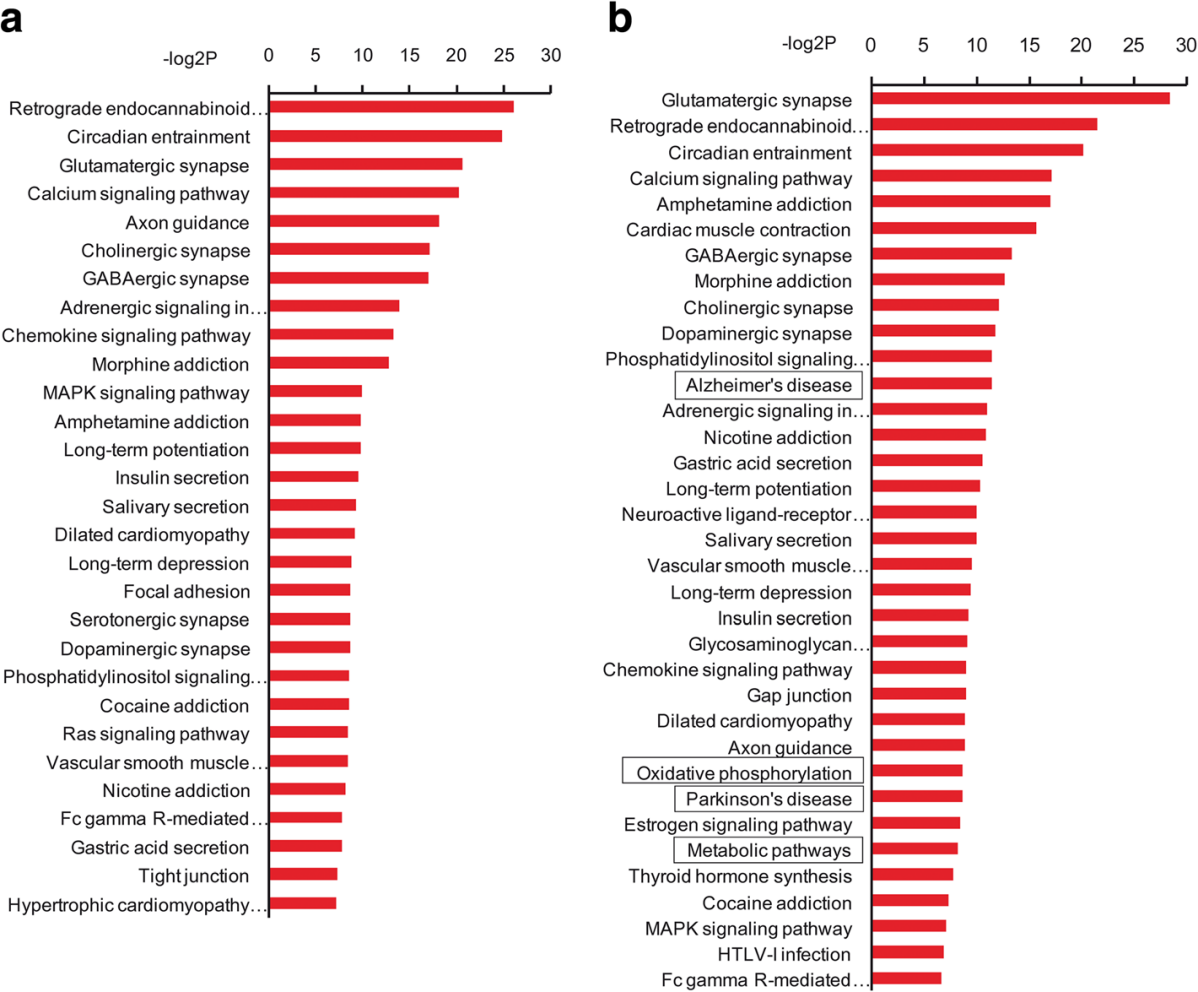
Pathways associated with DEGs between Sst- and Thy1- neurons in the absence and presence of nicotine. **a** In animals without nicotine treatment, the top ranked pathways associated with DEGs between Sst- and Thy1- neurons are depicted. **b** In mice treated with nicotine, the top ranked pathways associated with DEGs between Sst- and Thy1- neurons are present. Some new pathways including Alzheimer’s disease, Parkinson’s disease, oxidative phosphorylation and metabolic pathway are found in the presence of nicotine. Except for metabolic pathway, the DEGs are significantly overlapped between pathways and are associated with mitochondrial respiratory chain complex

**Fig. 5:**
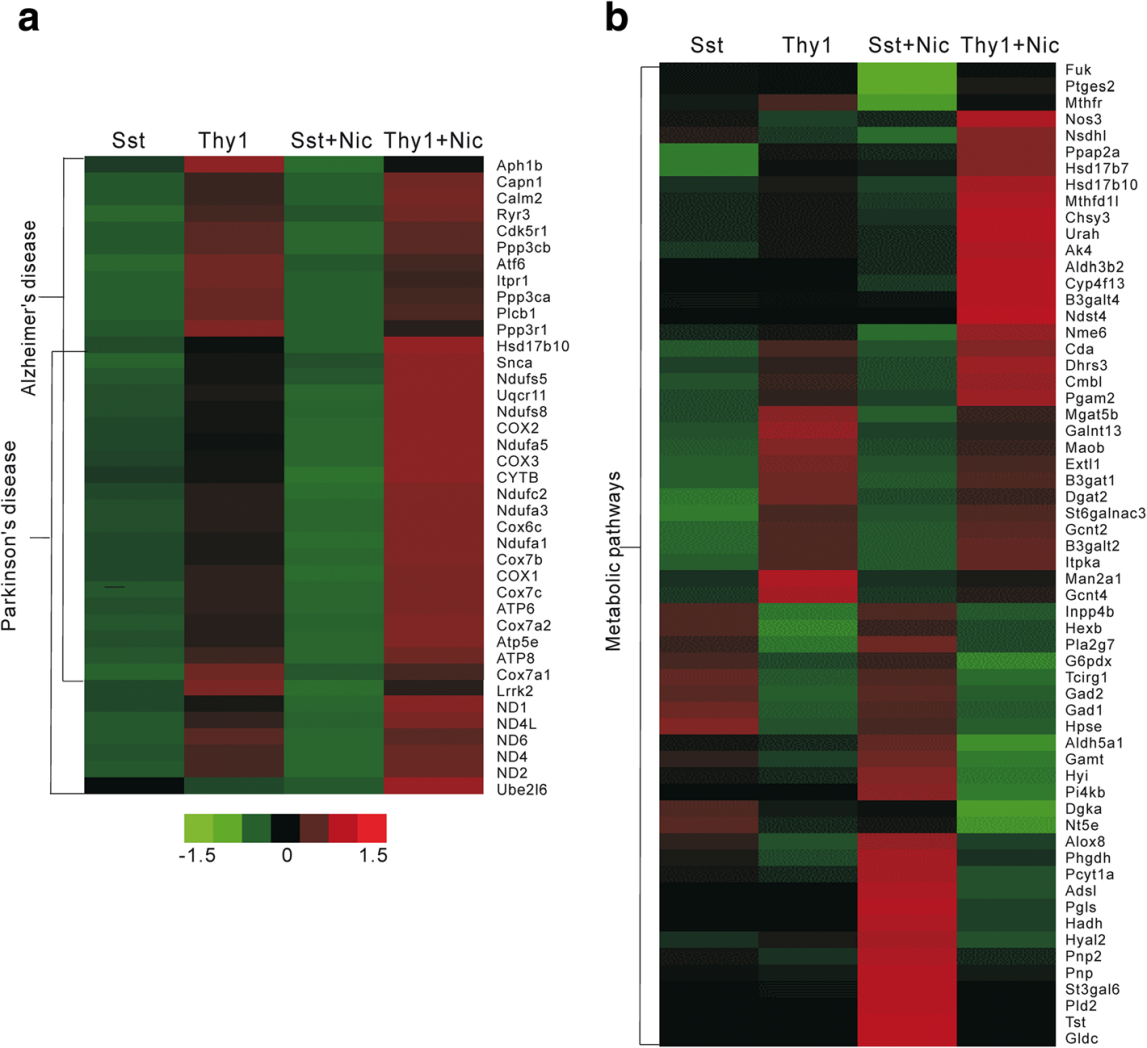
Representative genes in selected pathways. **a** The heat map of DEGs involved in Alzheimer’s disease (AD) and Parkinson’s disease (PD). Paired columns on the left: most of the genes in these pathways are relatively low in Sst-neurons and relatively high in Thy1-neurons with no significant difference in normal condition (Sst vs. Thy1, *P* > 0.05, *n* = 3). Paired columns on the right: the differences between Sst- and Thy1- neurons become significant after nicotine administration (Sst + Nic vs. Thy1 + Nic, *P* = 3.57E-4 for AD, *P* = 2.43E-3 for PD, *n* = 3). **b** The heat map of DEGs in metabolic pathway. Paired columns on the left: comparison of transcripts between Sst- and Thy1- neurons without nicotine (Sst vs. Thy1, *P* > 0.05, *n* = 3). Paired columns on the right: comparison of transcripts between Sst- and Thy1- neurons with nicotine (Sst + Nic vs. Thy1 + Nic, *P* = 3.37E-3, *n* = 3). Pseudocolor scale (−1.5 to 1.5): *green* represents low expression and *red* represents high expression

To further assess nicotine effect on DEGs between Sst- and Thy1-neurons, we performed pathway analysis according to KEGG data base in the absence and presence of nicotine (Additional file 9: Supplemental Excel S6). As shown in Fig. 4a & b, the DGEs between Sst- and Thy1-neurons associated with many common pathways remained exist regardless of nicotine application, which included retrograde endocannabinoid signaling, calcium signaling, axon guidance, glutamatergic synapse, cholinergic synapse, morphine addiction and GABAergic synapse, indicating that DEGs related to these pathways were not affected by nicotine. However, some new DEGs associated with Alzheimer Disease (AD, *P* = 3.57E-04), oxidative phosphorylation (*P* = 2.43E-03), Parkinson Disease (PD, *P* = 2.43E-03) and metabolic pathway (*P* = 3.37E-3) were present only in nicotine treated mice (marked by the rectangles in Fig. 4b). For most genes associated with AD and PD, significant changes were absent between Sst- and Thy1- neurons (Sst vs. Thy1, *P* > 0.05) in normal condition. After nicotine treatment however, these genes were significantly down-regulated in Sst-neurons compared with those in Thy1- neurons (Sst + Nic vs.Thy1 + Nic, Fig. 5a). Among these significantly changed genes, 62.5% in AD pathway and 74.1% in PD pathway were overlapped and were mitochondrial respiratory chain complex genes, which are closely associated with oxidative phosphorylation. In metabolic pathways, some top-ranked genes were shown in Fig. 5b. Different from AD/PD pathway genes which showed a clear trend of decrease (Sst vs. Thy1) in the presence of nicotine, metabolic pathway genes exhibited a complex profile. Both significantly decreased and increased genes in Sst neurons relative to Thy1 neurons were found after nicotine (Sst + Nic vs.Thy1 + Nic, Fig. 5b). Collectively, these results suggested that the relative alteration of mitochondrial respiratory chain complex genes between Sst- and Thy1- neurons may contribute to nicotinic regulation of neural network function, in which metabolic pathway may also play a role.

### Validation of representative genes by RT-PCR

**Fig. 6:**
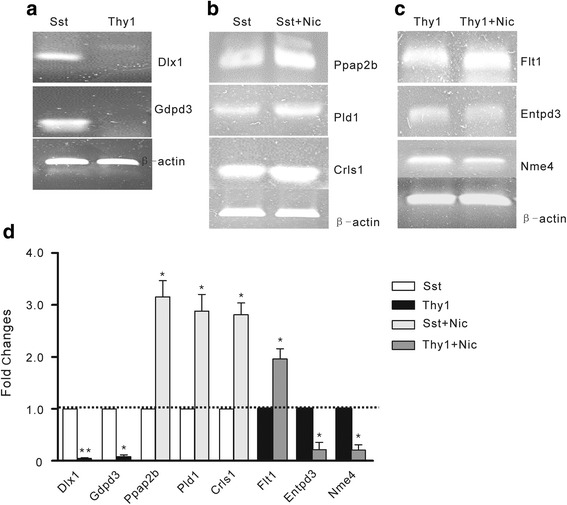
Semi-quantitative RT-PCR validation of representative DEGs. **a** Dlx1 and Gdpd3 were specifically expressed in Sst-neurons. **b** Increased expression of Ppap2b, Pld1 and Crls1 are shown in nicotine treated Sst-neurons. **c** Nicotine increases the expression of Flt1, and decreases that of Nme4 and Entpd3 in Thy1-neurons. **d** The densitometry analysis of the RT-PCR results from **a**, **b** & **c**. The expression levels of corresponding genes were normalized by β-actin. Data are shown in means ± SEM from at least three independent experiments (*P < 0.05, **P < 0.001, Student’s *t*-test)

To validate RNA-seq findings, we examined the expression level of some hub genes described in Figs. 1 & 2 by RT-PCR. As expected, the expression of Dlx1 and Gdpd3 were very low in Thy1-neuron relative to the high level in Sst-neurons. And Dlx1 that is necessary for interneuron differentiation and migration [45, 49] was selectively expressed in Sst- neurons (Fig. 6a). Consistent with RNA-seq findings, Ppap2b, Pld1 and Crls1 were significantly up-regulated by nicotine in Sst neurons. In addition, Flt1 was up-regulated and Entpd3 and Nme4 were down-regulated by nicotine in Thy1-neurons (Fig. 6b & c).

## Discussion

Previous microarray studies have shown that in SH-SY5Y cells, brief exposure (1 h) of nicotine (1 mM) results in DEGs associated with 14 pathways, in which the Toll-like receptor and death receptor pathways involved in immune response were significantly affected [19]. On the other hand, chronic nicotine causes regional DEGs, with prefrontal cortex (PFC) and nucleus accumbens (NAc) being most responsive to nicotine. The DEGs induced by nicotine in these areas are involved in phosotidylinositol signaling, calcium homeostasis and neuroprotection [50]. In addition, a comparative study reveals that genes involved in protein modification are altered by systemic nicotine in a region specific manner [21]. In the present study, we provide evidence that the effect of nicotine on DEGs is also dependent on distinct cell types. In addition, the relative DEGs between interneuron and pyramidal neurons might help understand how nicotine regulates brain functions at circuit level.

### Effect of nicotine on lipid metabolism in interneurons

In our work, Pld1 (phospholipase D1), Pld2 and Ppap2b (phosphatidic acid phosphatase 2b, LPP3) in interneurons are significantly increased by nicotine. These results are consistent with the notion that glycerophospholipid signaling is affected by nicotine [50]. Neuronal PLD can be activated by variety of stimulators such as neurotransmitters, hormones and growth factors [51]. The basic functions of PLD are associated with vesicular trafficking, brain development and neuroprotection [52]. However, different roles of PLD1 and PLD2 in Alzheimer Disease have been found. While PLD1 acts as a negative regulator of β-amyloid (Aβ) formation [53], PLD2 is required for Aβ-induced synaptotoxic action [54]. PPAP2b is a lipid phosphohydrolase enzyme that catalyzes the conversion of phosphatidic acid (PA) to diacylglycerol (DAG). It also hydrolyzes lysophosphatidic acid (LPA), ceramide-1-phosphate (C1P) and sphingosine-1-phosphate (S1P), thus participating in a variety of cellular signaling [55]. PPAP2b plays a key role in neuronal development through S1P signaling [56]. However, the function of PPAP2b in interneurons is poorly understood.

### Effect of nicotine on immune response and purine and pyrimidine metabolism in pyramidal neurons

An important pathway affected by nicotine is immune response. Two genes upregulated by nicotine are Csf1r (macrophage colony stimulating factor 1 receptor, M-CSFR) and Flt1 (fms-related tyrosine kinase 1, VEGFR1). CSF1R can be activated by CSF1 and interleukin-34 (IL-34), thus contributing to innate immunity by regulating the development of macrophage and microglia in the brain [57]. FLT1 regulates VEGF mediated angiogenesis [58]. It also binds to PlGF (placenta growth factor) that acts as a cytokine and is neuroprotective for cortical neurons [59, 60]. These results are consistent with the findings that nicotine regulates innate immune response in neuronal cells [19, 61]. The immune responses in the central nervous system are thought to modulate endocrine activity that controls cell migration, thermoregulation, drinking and feeding, among others [62]. Furthermore, the chemokine mediated neuo-glial crosstalk plays important role in multiple sclerosis and AD [63].

Systemic application of nicotine leads to down-regulated genes associated with purine and pyrimidine metabolism, in which Nme4 (nucleoside diphosphate kinase D, NDPKD) and Nt5e (5′-nucleotidase, ecto, CD73) are considered to be highly-connected genes. NME4 binds to mitochondrial inner membrane through cardiolipin and is associated with short chain fatty acids metabolism, Kreb cycle and apoptosis [64]. NT5E is a membrane-anchored protein that catalyzes the extracellular formation of adenosine from AMP. This protein mediates the inhibition of hippocampal synaptic plasticity and nociception [65, 66]. Although the functional consequences of these changes are not well understood, a recent study demonstrates that these genes are abnormally expressed in the brain of Parkinson disease [67].

### Intrinsic transcriptome differences between Sst- and Thy1- neurons

Our study demonstrates that many intrinsic differences between interneurons and pyramidal neurons remain exist regardless of nicotine administration. Consistent with previous reports [17, 32], the majority of DEGs between Sst- and Thy1- neurons in our study are associated with intracellular signal transduction, nervous system development and ion channel expression and regulation (Fig. 3). Pathway analysis links these genes to neurotransmitters (glutamate and GABA), neuromodulators (e.g. dopamine, serotonin and acetylcholine) and drug addiction relative to cocaine, morphine and nicotine (Fig. 4a), suggesting that these DEGs might contribute to the integrated function of cortical interneurons and pyramidal neurons [2]. In addition, we also find some new cell-specific genes, which include Thsd7a (thrombospondin, Type I, domain containing 7A) in Sst-neurons and Kcnh7 (potassium voltage-gated channel subfamily H member 7, HERG-3) and Crym (crystallin Mu, NADP-regulated thyroid-hormone-binding protein) in Thy1-neurons. The functional role of these genes is currently unknown.

### Nicotine induces relative DEGs associated with mitochondrial respiratory chain between Sst- and Thy1- neurons

Major functions of mitochondria in neurons include the regulation of synaptic plasticity [68, 69]. Our study demonstrates that chronic nicotine causes significantly decreased genes of mitochondrial respiratory chain in interneurons relative to pyramidal neurons, suggesting that nicotine favors pyramidal activity. Consistently, ectrophysiological studies have demonstrated that chronic exposure to nicotine results in persistent depression of interneuron, while glutamatergic neurotransmission is always increased [70, 71]. At circuit level, nicotine induces gamma oscillations in hippocampal neurons [72, 73], which are associated with the integrated function of pyramidal neurons and interneurons [74]. Thus, the relative DEGs between Sst- and Thy1- neurons might play a key role in nicotinic regulation of synaptic plasticity and network function. Unlike mitochondrial genes which show a clear trend of relative decrease in interneurons after nicotine, both the decreased and the increased genes are found in metabolic pathway, suggesting the complicated regulation. Example genes in metabolic pathway include nsdhl (NAD (P) dependent steroid dehydrogenase-like), mthfd1l (methylenetetrahydrofolate dehydrogenase (NADP^+^ dependent) 1-Like) and dhrs3 (dehydrogenase 3). However, the functional role of these genes in neurons is not well-understood.

## Conclusions

Is this study, most DEGs by nicotine are not enriched in Sst- or Thy1- neurons (FPKM <1000, Additional file 4: Supplemental Excel S2 & Additional file 5: Supplemental Excel S3), suggesting that nicotine might not have significant effect on major transcripts [30]. However, the relative changes of mitochondrial genes between Sst- and Thy1- neurons are highly enriched (FPKM >1000, Additional file 9: Supplemental Excel S6), which implies that nicotine may play a more prominent role in the regulation of functional balance between interneurons and pyramidal neurons. In addition, although many of the genes affected by nicotine can be categorized into pathways, still the majority of genes cannot be grouped, which does not rule out their functional importance. Moreover, the limitation of this study is that nicotinic effect was assessed from two genetically different mouse strains, thus the potential bias of DEG results between Sst- and Thy1- neurons may be existed (Fig. 3), as compared to the findings by Sugino K and colleagues [17]. Nonetheless, our study highlights the following findings: (1) Interneurons instead of pyramidal neurons might play a dominant role in nicotinic regulation of glycerophospholipid signaling in specific brain regions [50]. (2) Pyramidal neurons might be important in nicotinic regulation of immune response and calcium signaling [61, 75]. (3) The relative alterations of mitochondria related genes may contribute to nicotinic regulation of synaptic activity and neural network function [68, 73]. However, the role of the highly connected genes affected by nicotine in individual neurons is currently unknown, which remains to be studied in the future.

## Additional files

Additional file 1: Table S1. Paired primer sequences used for RT-PCR. **Table S2.** Clean Summary. **Table S3.** Mapping result statistics. **Table S4.** Gene expression levels associated with non-neurons. (DOC 101 kb)
Additional file 2: Figure S1. Overview of RNA-sequencing quality. A: Percentage of mapped reads onto the regions of exon, intron, 5′-untranslated region (5′-UTR), 3′-untranslated region (3′-UTR), transcription start site (TSS), transcriptionend site (TES) and intergenic region (InterGenic) in all samples. B-E: Distribution of reads on chromosomes in independent samples of Sst- and Thy1-neurons, in the absence (- Nic) and presence of nicotine (+ Nic). (TIF 8790 kb)
Additional file 3: List of DEGs affected by nicotine in Sst- and Thy1-neurons. (XLSX 203 kb)
Additional file 4: Pathways and related genes affected by nicotine in Sst neurons. (XLSX 33 kb)
Additional file 5: Pathways and related genes affected by nicotine in Thy1 neurons. (XLSX 23 kb)
Additional file 6: List of DEGs between Sst- and Thy1-neurons﻿ in the﻿ absence of nicotine. (XLSX 392 kb)
Additional file 7: GO analysis of DEGs between Sst- and Thy1-neurons in the﻿ absence of nicotine. (XLSX 76 kb)
Additional file 8: Figure S2. Heat map of top 80 cell-type-specific genes expressed in Sst- and Thy1-neurons. The genes with high expression pattern (FPKM > 1000, top and bottom) relative to low expression pattern (Fold change >20, FDR = 0) are selected. For low-expression genes, those with FPKM > 10 are shown on the top, while those with FPKM < 10 are shown on the bottom (*n* = 3). Gene symbols with expression pattern consistent with published observations are shown in red. (TIF 1891 kb)
Additional file 9: Comparisons of pathways in the absence and presence of nicotine. (XLSX 172 kb)

## Abbreviations

ACSF: Artificial cerebral spinal fluid
AMPA: α-amino-3-hydroxy-5-methyl-4-isoxazolepropionic acid
BSA: Bovine Serum Albumin
DEG: Differentially expressed genes
DNQX: 6, 7-dinitroquinoxaline-2,3-dione
FPKM: Fragments Per Kilobase of exon model per Million mapped reads
GABA: Gamma aminobutyric acid
KEGG: Kyoto encyclopedia of genes and genomes
nAChRs: Nicotinic acetylcholine receptors
NMDA: N-methyl-D-aspartic acid receptor
Sst: Somatostatin
Thy1: Thymus cell antigen 1
TTX: Tetradotoxin
