# Supplementary material for: Chronic nicotine differentially affects murine transcriptome profiling in isolated cortical interneurons and pyramidal neurons
Source: BMC Genomics. 2017 Feb 20;18:194. doi: 10.1186/s12864-017-3593-x (PMC5319194; doi:10.1186/s12864-017-3593-x)
Supplement: Additional file 1: Table S1. — Paired primer sequences used for RT-PCR. Table S2. Clean Summary. Table S3. Mapping result statistics. Table S4. Gene expression levels associated with non-neurons. (DOC 101 kb) [file 12864_2017_3593_MOESM1_ESM.doc]

## Table S1. Paired primer sequences used for RT-PCR

| **Gene** | **Primer sequence** | **Product size (bp)** |
| --- | --- | --- |
| **Pld1** | F：TATTGCCTTCGTCCTCCTTGTAG | 141 |
|  | R：CACCGGGCGTGTCTGTAG |  |
| **Dlx1** | F：ATGCCAGAAAGTCTCAACAGC | 118 |
|  | R：AACAGTGCATGGAGTAGTGCC |  |
| **Ppap2b** | F: GGCGATCGTCCCTGAGAGTAAGAA | 108 |
|  | R: AGGCAGAAGAGGTCCAGGCAGAT |  |
| **Csf1r** | F: GCCCGCCTGCCTGTAAAGTG | 132 |
|  | R: GCCGGGGTAGGGGTTCAGAC |  |
| **Flt1** | F: GCGCCACACCTGCTTCAAA | 92 |
|  | R: GGCGCGGGGACACCTCTA |  |
| **Nme4** | F: CAAGCTCGTGGGGATGAAG | 99 |
|  | R: CTGATAAGAGCTGGGTAGAATGGC |  |
| **Entpd3** | F: GCCAGTGCGCTAAACCTCTCAG | 130 |
|  | R: ACCGGGCATACGTCTCATCAAAT |  |
| **Gdpd3** | F:TAGGCGCTTTGACCGGAATGAGAT | 152 |
|  | R:GGAGCAGCCCCAGGTAGTAGAGCA |  |
| **Crls1** | F:TCTGCCAACACCGCGAACACTA | 127 |
|  | R:TGCCAAAGAAGCTGCCACCAA |  |
| **Ndufa3** | F:CTGGTGGTGTCCTTCTCTGTC | 109 |
|  | R:CTGGGTAGTTGTAGGGTGTGG |  |
| **Ndufa5** | F:TGCGACACTCCACACGA | 139 |
|  | R:GCTCCGCCTTGACCATA |  |
| **Cox1** | F:TACTAACAGACCGCAACCTAAACA | 94 |
|  | R:TGCCCAAAGAATCAGAACAGA |  |
| **ATP6** | F:GGCCTTTTACCACATACATTTACA | 84 |
|  | R:TACGGCTCCAGCTCATAGTG |  |
| **β-actin** | F:CACGATGGAGGGGCCGGACTCATC | 240 |
|  | R:TAAAGACCTCTATGCCAACACAGT |  |

**Table S2.** Clean Summary

| **Sample ID** | **Good reads** | **Quality trimed** | **Adaptor**  **trimed** | **Clean reads** | **rRNA**  **trimed** | **Clean**  **ratio(%)** |
| --- | --- | --- | --- | --- | --- | --- |
| **9923** | 61503054 | 60777822 | 59874920 | 58344200 | 58168590 | 94.58 |
| **9929** | 40918200 | 40339686 | 39726748 | 38588978 | 38500372 | 94.09 |
| **9937** | 45808478 | 45300315 | 44631537 | 43519900 | 43397392 | 94.74 |
| **9950** | 46289718 | 45593354 | 44916654 | 43619046 | 43446994 | 93.86 |
| **9952** | 51726042 | 51027729 | 50259713 | 48901476 | 48708302 | 94.17 |
| **9958** | 44834120 | 43982201 | 43291386 | 41835904 | 41324412 | 92.17 |
| **9959** | 48421584 | 47788036 | 47089025 | 45825462 | 45629180 | 94.23 |
| **9971** | 67842486 | 66859770 | 65835075 | 63930380 | 63579270 | 93.72 |
| **9972** | 48182152 | 47451896 | 46549442 | 45238168 | 45134830 | 93.68 |
| **9973** | 46222194 | 45375706 | 44671611 | 43198568 | 43003450 | 93.04 |
| **9975** | 49303834 | 48493417 | 47741273 | 46256020 | 46045162 | 93.39 |
| **9976** | 50019942 | 49090085 | 48319882 | 46703176 | 46557398 | 93.08 |

**Note**: The ID numbers are corresponding to the following neuron groups. 9923, 9975, 9976 for SST; 9973, 9937, 9971 for Sst+Nic; 9972, 9959, 9950 for Thy1; and 9958, 9952, 9929 for Thy1+Nic, respectively.

**Table S3.** Mapping result statistics

| **Samples_ID** | **All reads** | **Mapped reads** | **Mapped**  **Pair**  **reads** | **Mapped**  **broken-pair**  **reads** | **Mapped**  **Unique**  **reads** | **Mapped**  **Multi**  **reads** | **Mapping ratio(%)** |
| --- | --- | --- | --- | --- | --- | --- | --- |
| **9923** | 58168590 | 45537473 | 44062156 | 1475317 | 39665368 | 5872105 | 78.29 |
| **9929** | 38500372 | 30753719 | 29908164 | 845555 | 26837489 | 3916230 | 79.88 |
| **9937** | 43397392 | 32849692 | 31737442 | 1112250 | 29185305 | 3664387 | 75.7 |
| **9950** | 43446994 | 34101711 | 33145716 | 955995 | 29950621 | 4151090 | 78.49 |
| **9952** | 48708302 | 39817209 | 38763068 | 1054141 | 34155776 | 5661433 | 81.75 |
| **9958** | 41324412 | 30301386 | 29333592 | 967794 | 25396606 | 4904780 | 73.33 |
| **9959** | 45629180 | 36473054 | 35432820 | 1040234 | 32341833 | 4131221 | 79.93 |
| **9971** | 63579270 | 48084830 | 46415114 | 1669716 | 42345343 | 5739487 | 75.63 |
| **9972** | 45134830 | 22441330 | 21595304 | 846026 | 19334042 | 3107288 | 49.72 |
| **9973** | 43003450 | 31224980 | 30119270 | 1105710 | 26384173 | 4840807 | 72.61 |
| **9975** | 46045162 | 33858778 | 32637158 | 1221620 | 29186022 | 4672756 | 73.53 |
| **9976** | 46557398 | 33756868 | 32531740 | 1225128 | 28957905 | 4798963 | 72.51 |
| **9923** | 58168590 | 45537473 | 44062156 | 1475317 | 39665368 | 5872105 | 78.29 |
| **9929** | 38500372 | 30753719 | 29908164 | 845555 | 26837489 | 3916230 | 79.88 |

**Note**: The ID numbers are corresponding to the following neuron groups. 9923, 9975, 9976 for SST; 9973, 9937, 9971 for Sst+Nic; 9972, 9959, 9950 for Thy1; and 9958, 9952, 9929 for Thy1+Nic, respectively.

## Table S4. Gene expression levels associated with non-neurons

| **Gene** | **Locus** | **Thy1 neuron-1** | **Thy1 neuron-2** | **Thy1 neuron-3** | **Sst neuron-1** | **Sst neuron-2** | **Sst neuron-3** |
| --- | --- | --- | --- | --- | --- | --- | --- |
| **Vim** | gia | 2.43 | 0.00 | 0.00 | 0.00 | 5.98 | 0.00 |
| **Slc1a3** | glia | 3.89 | 0.00 | 0.67 | 0.41 | 4.07 | 3.03 |
| **Metrn** | gila | 0.00 | 0.00 | 0.00 | 0.62 | 0.00 | 0.00 |
| **Gfap** | astrocyte | 0.56 | 0.00 | 0.00 | 0.00 | 3.53 | 0.00 |
| **Aqp4** | astrocyte | 0.08 | 0.00 | 0.14 | 0.00 | 1.00 | 0.00 |
| **Mfge8** | astrocyte | 2.76 | 0.00 | 0.19 | 0.05 | 1.10 | 0.03 |
| **Olig1** | oligodendrocyte | 0.05 | 0.00 | 5.37 | 2.63 | 1.82 | 2.06 |
| **Itpr2** | oligodendrocyte | 0.00 | 0.00 | 0.00 | 0.02 | 0.17 | 0.09 |
| **Aif1l** | microgila | 1.30 | 0.00 | 2.74 | 0.00 | 1.10 | 0.00 |
| **Hbb-b1**  **Hba-a1** | red blood cell   red blood cell | 0.00  1.99 | 0.00  0.63 | 0.00  0.00 | 0.00  0.00 | 0.00  0.06 | 0.00  0.00 |
| **Hba-a2** | red blood cell | 3.10 | 1.26 | 0.00 | 0.00 | 0.06 | 0.00 |

Note: The numbers are expressed as FPKM (Fragments Per Kilobase of exon model per Million mapped reads). FPKM<10 is considered as low expression, while FPKM≥ 10 is considered as high.
