# Supplementary figures and images for: Chronic nicotine differentially affects murine transcriptome profiling in isolated cortical interneurons and pyramidal neurons
Source: BMC Genomics. 2017 Feb 20;18:194. doi: 10.1186/s12864-017-3593-x (PMC5319194; doi:10.1186/s12864-017-3593-x)

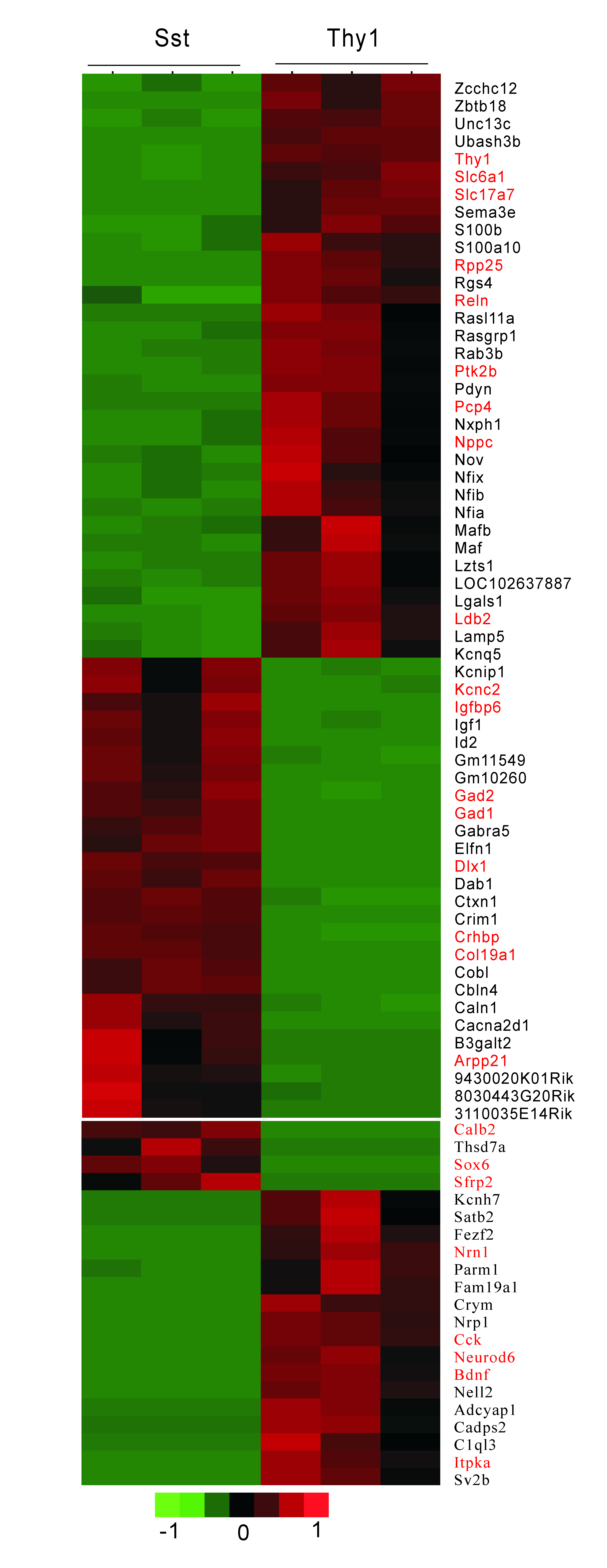

Supplement: Additional file 8: Figure S2. — Heat map of top 80 cell-type-specific genes expressed in Sst- and Thy1-neurons. The genes with high expression pattern (FPKM > 1000, top and bottom) relative to low expression pattern (Fold change >20, FDR = 0) are selected. For low-expression genes, those with FPKM > 10 are shown on the top, while those with FPKM < 10 are shown on the bottom (n = 3). Gene symbols with expression pattern consistent with published observations are shown in red. (TIF 1891 kb) [file 12864_2017_3593_MOESM8_ESM.tif]
